# Fuzzy sets allow gaging the extent and rate of species range shift due to climate change

**DOI:** 10.1038/s41598-020-73509-y

**Published:** 2020-10-01

**Authors:** Darío Chamorro, Raimundo Real, Antonio-Román Muñoz

**Affiliations:** Biogeography, Diversity, and Conservation Research Team, Department of Animal Biology, Science Faculty, Universidad de Málaga, 29071 Malaga, Spain

**Keywords:** Ecology, Biodiversity, Biogeography, Climate-change ecology, Conservation biology, Macroecology, Theoretical ecology

## Abstract

The recent modification of species distribution ranges in response to a warmer climate has constituted a major and generalized biogeographic change. The main driver of the shift in distribution is the disequilibrium of the species ranges with their climatic favourability. Most species distribution modelling approaches assume equilibrium of the distribution with the environment, which hinders their applicability to the analysis of this change. Using fuzzy set theory we assessed the response to climate change of a historically African species, the Atlas Long-legged Buzzard. With this approach we were able to quantify that the Buzzard’s distribution is in a latitudinal disequilibrium of the species distribution with the current climate of 4 km, which is driving the species range northwards at a speed of around 1.3 km/year, i.e., it takes 3 years for the species to occupy new climatically favourable areas. This speed is expected to decelerate to 0.5 km/year in 2060–2080.

## Introduction

Environmental, biological, historical, and anthropogenic factors affect the distribution of species in space and time^1^. However, climate seems to be the most relevant factor for several taxa at large scales^2–6^. Throughout the last century, and especially over the last few decades, there have been global alterations leading to a warmer climate^7^ with widespread effects on biological systems^8–10^. This recent climate change has already caused expansions, reductions, or shifts in the distribution of many species^11–14^. In particular, climate shifts are modifying the margins of species distributions over short periods of time, a process that is more marked in vagile species such as birds^15,16^.

In general, it is only in recent decades that studies have addressed the reaction of birds to climate change in relation to modifications in their distribution^17–19^ and phenology^20–22^. These modifications are relevant not only for the species undergoing the changes, but also for the species residing in the new receiving areas^14^. Distribution changes ultimately affect community composition, which adds uncertainty to the future status of natural populations and forces changes in species management and conservation programs^23,24^.

The Strait of Gibraltar, 14 km at its narrowest, separates Africa and Europe at the same time that puts the two continents in close contact. It is a difficult-to-cross bridge for many migratory species, especially soaring birds^25–27^, and is also an effective biogeographic barrier for many other taxa^28–30^. However, this barrier is currently being overcome by some typical African birds: that is, taxa that until very recently had their northern distribution limit in Northern Africa^31–34^. These changes are putting into contact two faunas in the European part of the Strait of Gibraltar, entailing changes in communities that are leading to new ecological interactions in this region^35^. The southern part of the Iberian Peninsula is a suitable place for colonization by different African bird species^36^, for which it is beginning to act as a focal point in the colonization of Europe^2,37^. In order to forecast these distribution ranges, more information is needed on whether these species are expanding or shifting their distributions, the spatial and temporal disequilibrium between the species ranges and their climatic favourability, the latitudinal rate of their change in distribution range, and whether these rates are expected to increase, decrease or remain constant in the future.

**Figure 1:**
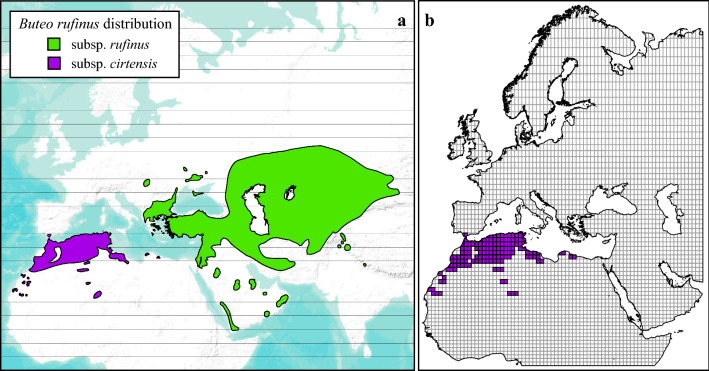
(**a**) *Buteo rufinus* distribution separated by the two accepted subspecies, modified from the IUCN shapefile (www.iucnredlist.org). (**b**) Study area 100 × 100 km grid cell (the presences used for the modelling process are shown in violet). The maps were created using ArcMap software (ArcGIS 10.4.1) https://desktop.arcgis.com/es/arcmap/.

The long-legged buzzard is an example of a species on the move. Traditionally distributed from Mauritania and Morocco eastwards to Egypt (Fig. 1a)^38–41^, the Atlas long-legged buzzard (*Buteo rufinus cirtensis*), has recently colonized Europe^19^. Chamorro et al.^2^ conducted a biogeographic study of this subspecies and showed that climate is the most important factor affecting the distribution of the Atlas Long-legged Buzzard, suggesting that climate change is the main driver of this biogeographic change. This assessment was based on the notion of climatic favourability, a typical fuzzy concept whose theoretical foundation rests on fuzzy set theory rather than on niche theory. A related subspecies, the Asian Long-legged Buzzard (*Buteo rufinus rufinus*), breeds from the Balkans and Asia Minor eastwards to Mongolia and northwest India (Fig. 1a)^42^.

Since the beginning of the 2000s, fuzzy set theory and its corresponding fuzzy logic^43^ have spread from the fields of engineering, finances or psychology and increasingly used in biogeography (e.g. Refs.^44–47^). A fuzzy set is a class of objects with a continuum of degrees of membership, such that the set is defined by a membership function which assigns each object with a value ranging from zero to one^43^. Fuzzy sets have no sharply defined boundaries and so they better reflect the continuous character of nature^48^. Thus, fuzzy logic provides a conceptual framework that has a particular scope of applicability in the domains of biogeographic pattern classification and information processing. It is more successful than classical approaches to managing typical fuzzy notions such as the presence of a species in a particular region, the identification of climatically favourable areas for reproduction, or climatic similarity between localities^43,49,50^.

In this paper, we use fuzzy logic methods to analyse the dynamics of species distribution in the context of climate change, quantifying the extent of colonization as well as displacement rates in climatically favourable areas. We applied these methods to assess the favourability of the Western Palearctic for the Atlas Long-legged Buzzard, and to determine if the species is actually being pushed northward due to climate change. We forecast its response to future climate change scenarios (i.e., whether its distribution will expand and at what rate). Thus, we attempt to provide information on the ongoing and future occupation of Europe and the northern Mediterranean basin by African birds. This information will be of interest for the environmental management of potentially affected areas (Supplementary Information).

## Results

**Figure 2:**
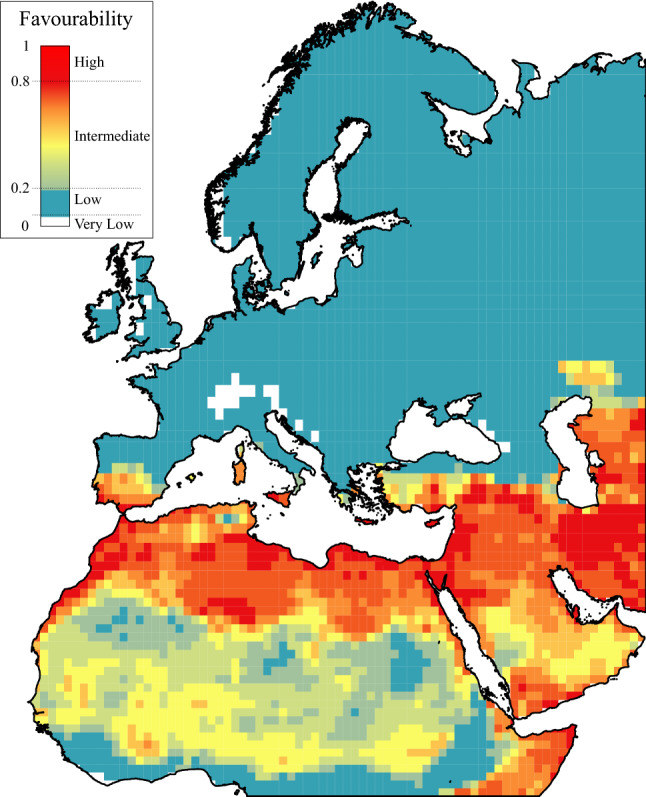
Cartographic representation of the current climatic favourability for Atlas Long-legged Buzzard breeding in each OGU of the study area. The mathematical model is shown in Table 1. The map was created using ArcMap software (ArcGIS 10.4.1) https://desktop.arcgis.com/es/arcmap/.

The climatically favourable areas for the Atlas Long-legged Buzzard include Northern Africa, Sicily, the Middle East, and the Southwest of the Iberian Peninsula. The latter area is the only continental European region where the climate is highly favourable (Fig. 2). In Africa, highly favourable areas are located in Morocco, the Mediterranean coasts of Algeria, Tunisia, Egypt, and two inland areas of Algeria (Tassili n'Ajjer national park and El Menia oasis) (Figs. 1b, 2). In the Middle East, favourable areas are in the margins of the Mediterranean and Caspian seas, the Arabian Peninsula, Cyprus, Iran, and Turkey. These areas are currently occupied by its sister subspecies, the Asian Long-legged Buzzard (Figs. 1a, 2).

The climatic favourability model included six variables (Table 1). A small diurnal temperature range, cool temperatures during the wettest season, and scarce precipitation in the driest month are the best predictors for Atlas Long-legged Buzzard breeding because these variables were the first to be entered in the stepwise procedure. In addition, under the Wald test, these variables have the most weighting in the model (Table 1).

**Table 1:** Variables entered in the logistic regression model by the forward–backward step-wise selection process, ranked by their order of entrance.

Variable	β	Wald	*p*
*PrecDryMonth*	− 0.2199	29.406	5.86 × 10^–08^
*TempWetQ*	− 0.1212	34.327	4.65 × 10^–09^
*DiTempRange*	− 0.4175	44.148	3.04 × 10^–11^
*Prec*	0.0013	13.103	2.94 × 10^–04^
*MeanTemp*	0.0980	7.874	5.01 × 10^–03^
*Alti*	0.000477	5.603	1.79 × 10^–02^
Constant	3.4712	20.707	5.35 × 10^–06^

β are the coefficients in the *logit* function, Wald is the Wald’s statistics value (representing the relative importance of the variable in the model) and *p* the significance of the coefficients. Codes of the variables are the same as in Table 5.

**Figure 3:**
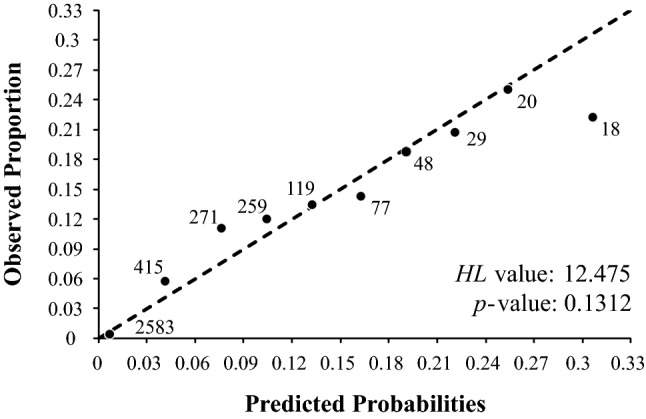
Graphic representation of the Hosmer and Lemeshow test values for each bin, with the number of cases at each bin.

The model had high classification power (Table 2), higher sensitivity than specificity, and a high over-prediction rate (OPR > 0.8). Discrimination capacity was high (AUC > 0.8) and highly significant (*p* = 6.516 × 10^–51^). The Pch value of the model was remarkably high (Table 2). The Hosmer and Lemeshow calibration test (Fig. 3) showed that differences between expected and predicted values were nonsignificant (*p* > 0.05) over the whole range of expected probability values. Good calibration is very difficult to obtain when there are many operational geographic units (OGUs) because small relative differences between observed and predicted probabilities generate significant values in the statistic^51^.

**Table 2:** Assessment indices: Prevalence of the model (*n*_1_/*n*), area under the curve (AUC), Cohen’s Kappa, sensitivity, specificity, correct classification rate (CCR), under-prediction rate (UPR), over-prediction rate (OPR), and factor of potential change (Pch).

Measure	Value
Prevalence	0.03701
AUC	0.86542
Kappa	0.15271
Sensitivity	0.90411
Specificity	0.73941
CCR	0.74550
UPR	0.00496
OPR	0.88235
Pch	7.68493

**Figure 4:**
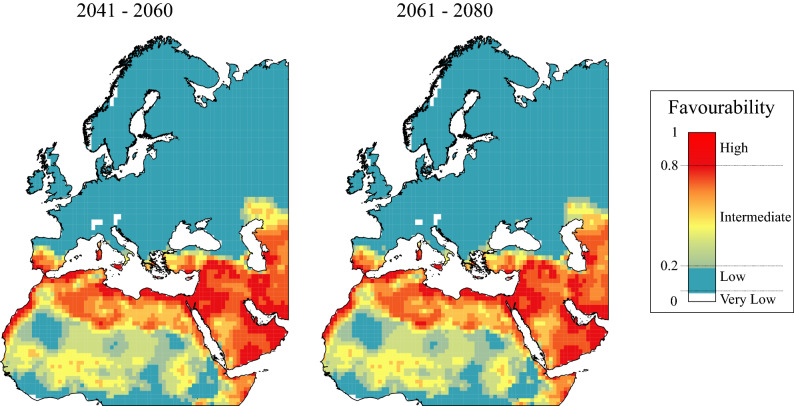
Atlas Long-legged Buzzard ensemble climatic favourability models for future periods of time. Maps created using ArcMap software (ArcGIS 10.4.1) https://desktop.arcgis.com/es/arcmap/.

Although forecast favourable areas did not differ substantially between the two periods of time, they differed from the present climatic favourability pattern (Fig. 4). Current breeding areas in North Africa are expected to have lower favourability values in the future, but other areas are expected to increase their favourability values in Sicily, Sardinia, and the Iberian and Arabian peninsulas (Figs. 2, 4).

**Figure 5:**
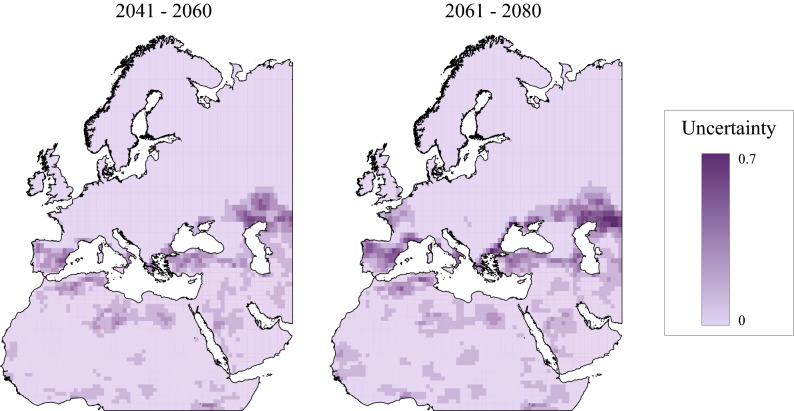
Climatic uncertainty for each period of time, associated with the different climate change scenarios analysed. Maps created using ArcMap software (ArcGIS 10.4.1) https://desktop.arcgis.com/es/arcmap/.

There was a low mean uncertainty of less than 0.1 associated with climate scenarios (Fig. 5). For the period 2061–2080, there is an increase in uncertainty values in some areas, such as on the Atlantic coast of France and the northern coast of the Black Sea, where in the period 2041–2060 the uncertainty values are much lower. The highest uncertainty values are in the northern half of the Iberian Peninsula, the northern edge of the Caspian Sea, the coast of the Balkan Peninsula, and Turkey, particularly for the period 2061–2080. Low uncertainty values are found in the south of the Iberian Peninsula and the coasts of north-western Africa.

A northward disequilibrium of 0.039 degrees of latitude was detected between the latitudinal barycentre and the climatic favourability barycentre of the OGUs with reported breeding of the buzzard (Table 3). A latitudinal degree is roughly equivalent to 111.12 km, and so this disequilibrium was equivalent to 4.3 km. Applying the same equivalence, the FD_20-60_ rate value was 1.3 km/year. If this rate is assumed to be similar to the current one, then the latitudinal disequilibrium of 4.3 km between distribution and climatic favourability is equal to a temporal disequilibrium of a little more than 3 years. The FD_60–80_ value was lower at 0.6 km/year.

**Table 3:** Results of the latitudinal variation assessment in decimal degrees.

Measure	Value
*B*_*pres*_	29.706
*B*_*FB*_	29.745
*B*_*FLo*_	25.388
*B*_*F60*_	25.868
*B*_*F80*_	25.971
*Ldis*	0.039
*FD*_*20–60*_	0.012
*FD*_*60–80*_	0.0051

Latitudinal barycentre of the actual breeding area (*B*_*pre*s_). Latitudinal climatic favourability barycentres of the OGUs with reported breeding of the buzzard (*B*_*FB*_), latitudinal climatic favourability barycentres of the OGUs inside the longitudinal range, where the subspecies was reported to breed, for the current model (*B*_*Lo*_), and the 2041–2060 (B_F60_) and the 2061–2080 (B_F80_) future ensemble forecasting models. Latitudinal disequilibrium between current climatic favourability for breeding and actual breeding (Ldis) and average rates of latitudinal climatic Favourability Displacement in decimal degrees per year for the 2041–2060 (FD_20–60_) and the 2061–2080 (FD_60–80_) future periods.

Table 4 shows the fuzzy logic indicators of other expected effects of climate change for each possible scenario and the ensemble forecast at each period of time. Increment values are low (I < 0.08) but generally positive: however, some negative values are expected in lower CO_2_ emissions scenarios (2.6, 4.5, and 6.0). Overlap values of present and future favourable areas are high (O > 0.78). Maintenance values for currently favourable areas are not expected to be complete (0.92 at most) although they are generally high (M > 0.87). In all cases, shift values are lower than 0.11 but higher than 0.08, indicating that between 8 and 11% of currently favourable areas for breeding are expected to be lost but could be replaced by new opportunities for breeding in emergent favourable areas elsewhere.

**Table 4:** Fuzzy logic indicators of the impact of climate change for each Global Circulation Model (GCM) and Representative Concentration Pathway (RCP) and the ensemble forecasting at each period of time: Increment (*I*), Overlap (*O*), Maintenance (*M*), Shift (*S*) and the cardinality of the favourability values for the future (*c*F_*f*_).

Time period	GCM	RCP	*I*	*O*	*M*	*S*	*c*F_*f*_
2041–2060	HadGEM2–ES	2.6	− 0.00198	0.818	0.899	0.0988	1,225.341
		4.5	0.0260	0.815	0.910	0.0903	1,259.751
		6.0	0.00871	0.822	0.906	0.0938	1,238.474
		8.5	0.0470	0.816	0.920	0.0804	1,285.514
	NorESM1–M	2.6	0.00845	0.817	0.903	0.0966	1,238.160
		4.5	− 0.00265	0.804	0.890	0.107	1,224.520
		6.0	− 0.000816	0.807	0.893	0.106	1,226.776
		8.5	0.0251	0.808	0.905	0.0947	1,258.708
	Ensemble		0.0137	0.821	0.908	0.0920	1,244.647
2061–2080	HadGEM2–ES	2.6	− 0.0155	0.821	0.895	0.0895	1,208.728
		4.5	0.0213	0.807	0.903	0.0972	1,254.035
		6.0	0.0190	0.808	0.902	0.0978	1,251.171
		8.5	0.0783	0.785	0.914	0.0859	1,324.008
	NorESM1–M	2.6	− 0.0279	0.817	0.887	0.0852	1,193.483
		4.5	− 0.0189	0.804	0.883	0.0979	1,204.510
		6.0	− 0.0239	0.799	0.878	0.0983	1,198.398
		8.5	0.01146	0.801	0.896	0.104	1,245.755
	Ensemble		0.00589	0.815	0.901	0.0994	1,235.0021

## Discussion

Five of the six significant predictive variables included in the favourability model were associated with climate (Table 1). In addition, the only non-climatic variable, altitude, was the last to be included in the model and had the lowest weight in the model under the Wald test criterion (Table 1), suggesting a finer-scale effect. Consequently, the favourability model can be rightly considered to be a climatic favourability model. The requirements of this buzzard are matched with Mediterranean climatic conditions^2^ as shown by the concentration of highly favourable areas for breeding in the Mediterranean basin and the generally low climatic favourability values in the European continent, which are especially low in the Alps, the Caucasus Mountains, and the British and Norwegian coasts. The main favourable climatic conditions included in our model are low precipitation during summer, low temperatures during the wettest season and stability in daily temperatures (Table 1). Especially in Europe, climate change is expected to increase the temperatures during day and night and to reduce the number of cold nights as well as increasing the frequency and duration of heat waves^7^, which will get the European region a warmer and drier environment. Extreme low temperatures, unlike extreme hot temperatures, are predicted to be less frequent during daily and seasonal timescales^7^, so despite the fact that stability in daily temperatures is considerably hard to foresee, climate change predictions suggest a low increment in the diurnal range of temperatures, except in the southern part of the study area. The annual mean precipitation will likely decrease in Europe, although in some humid regions it is predicted to slightly increase under some scenarios^7^. Under these conditions the possibility of establishment in Europe will likely increase in the coming years. The positive coefficient associated with altitude (Table 1) may be due to the fact that this species mainly nests in cliffs^52^. However, in the Iberian Peninsula it nests in trees^19,35^, which shows the plasticity of the species when choosing a nesting substrate. This behavioural change in nesting requirements could be mediated by a compensation of climatic favourability in areas where cliff-nesting areas are scarce or already occupied by other species. Such plasticity in nesting behaviour has also been demonstrated for other species^53^. This topographical variable could also be indicative of climatic conditions that are closely correlated with altitude^54^.

As our models were based on fuzzy set theory, they were able to measure the spatial and temporal disequilibrium between the buzzard breeding area and the climatic favourability for breeding. The mean climatically favourable condition occurs 4.3 km northwards of the mean latitudinal range of the buzzard breeding areas. The mere existence of this spatial disequilibrium demonstrates that, in order to be sound, favourability distribution models do not have to assume that the species must be in equilibrium with their environment, as models based on niche theory have customarily assumed^55,56^. In fact, a spatial disequilibrium is needed for the climatic conditions of the favourable areas to attract the buzzard and lead to changes in its breeding range^49,50,57^. The FD_20–60_ value suggests that the buzzard range is not in temporal equilibrium with the current climate by a margin of about 3 years. This result suggests that, if climatic conditions stop changing, the buzzard range could attain equilibrium with the climate in approximately 3 years. Otherwise, the effect of the climatic disequilibrium will continue to attract the breeding range to the favourable adjacent areas. This latitudinal range shift is expected to decrease to 0.6 km/year from 2060 to 2080, suggesting that the colonization of the Western Palearctic will decelerate according to current climatic projections.

Fuzzy logic operations (IOMS framework, Table 4) indicate that the climatic favourability for the Atlas Long-legged Buzzard is shifting rather than broadening. An initial indicator of this change is that the Increment values are low and even negative in some cases. This result would not be expected if the expansion of the range was northward, as this would include the maintenance of favourable areas in their southern range and the addition of new favourable areas in the north^13^. Maintenance values of only about 90% are mainly explained by the favourability loss in the southern limit of the current distribution, as in the Sahel region (Fig. 4). However, the main indicator is that the Shift values are more than zero, and practically identical to the part of the current favourability area that is not expected to be maintained (Table 4). This result indicates that the loss of climatically favourable areas in the south are expected to be compensated for by the appearance of new favourable areas in the north, particularly in the coast of northern Morocco, the southern half of the Iberian Peninsula, as other study previously stated^2^, and the Mediterranean islands of Sardinia and Sicily (Fig. 4). This loss may effectively push the breeding distribution of the buzzard northward. High Overlap between present and future climatic favourability models indicates that the current and expected favourable areas are geographically close^23^, which could facilitate the movement of buzzards to the newly available zones that are currently unoccupied. There is a high degree of similarity between all predicted future scenarios. The forecasts for the 2061 to 2080 period have the highest uncertainty, which is normal when the forecast time period is further away from the present^7^.

It should be noted that the Asian Long-legged Buzzard is currently occupying areas in the Middle East that have been identified as highly favourable for the Atlas Long-legged Buzzard^58^. This situation is likely preventing the establishment of the Atlas Long-legged Buzzard because of possible competitive exclusion^59–61^. It may also be the main reason for the high over-prediction rate and high Pch of the climatic favourability model for the Atlas Long-legged Buzzard (Table 2, Fig. 2), and that the range shift is more likely to be northwards rather than eastwards. This forecast is supported by empirical data, such as the increasing number of records of the Atlas Long-legged Buzzard in northern Spain^62–64^ and Portugal^65^ and its successful breeding since 2009 in the south of the Iberian Peninsula^19^.

However, other factors could interfere in the northwards colonization process, by delaying or even stopping the Atlas Long-legged Buzzard dispersion. For instance, hybridization with the Common Buzzard (*B. buteo*) has already been detected in Pantelleria Island (Italy) and southern Spain^35,66^. These areas represent new contact zones where these closely related species currently meet. Hybridization in these areas is favoured because the Atlas Long-legged Buzzard is currently rare and, hence, its choice of mate is restricted. If the offspring were infertile, hybridisation would be acting as a synecological barrier against the expansion of the Atlas Long-legged Buzzard into Europe^35,67^. Thus, some new climatically favourable areas could remain unoccupied by the Atlas Long-legged Buzzard in the future. It is therefore relevant to track its northward expansion into Europe by monitoring favourable areas on the distribution fronts, such as the Strait of Gibraltar, and in those areas detected as highly favourable in this study.

Many other African birds have already experienced or are currently experiencing a similar expansion pattern in the Iberian Peninsula. Some examples are the White-rumped Swift (*Apus caffer*), the Little Swift (*Apus affinis*), and the Common Bulbul (*Pycnonotus barbatus*), which is the most recent addition to the European avifauna and is already breeding^2,68–70^. Moreover, in the last decade, there has been an increasing number of reports of other African birds such as Rüppell’s Vulture (*Gyps rueppellii*), Lanner Falcon (*Falco biarmicus*), Cream-coloured Courser (*Cursorius cursor*), Moussier’s Redstart (*Phoenicurus moussieri*), and House Buntings (*Emberiza sahari*)^33,36,62,71^. Other species, such as the White-backed Vulture (*Gyps africanus*) and Bateleur (*Terathopius ecaudatus*) have also been recently reported in Europe for the first time^33,34^. These reports may indicate that European Mediterranean areas, and particularly the Iberian Peninsula, should be prepared for the establishment of new African fauna in the near future.

These species could use these areas as stepping stones to expand into the rest of Europe. For example, this pattern was followed by the Black-shouldered Kite (*Elanus caeruleus*), which started breeding in Tarifa (southern Spain), then moved northwards, and became established in France in 2013^69,72–74^. Some typical Mediterranean birds are also moving northwards and reaching Central Europe, where they are breeding for the first time. This is happening, for example, in Switzerland, where typical Mediterranean species, such as the Short-toed Snake-eagle (*Circaetus gallicus*) and the European Bee-eater (*Merops apiaster*) have settled recently and experienced population growth^75^. These observations indicate that a change in species composition is already occurring in Western Europe due to climate change.

Favourability models have proven to be useful tools to identify new potential areas for the arrival and establishment of new individuals in the near future^76,77^. The practical usefulness of these tools must be coupled to a sound theoretical background^78^. Our results reflect the interaction between the Atlas Long-legged Buzzard and its environment within a dynamic biogeographic framework. The favourability function is well established in fuzzy set theory and it also represents the response function of the species to the environmental conditions^44,54,79,80^. This aspect may constitute a contact point between fuzzy set theory and niche theory. In our view, this contact point requires adding notions of graduality to the niche concept, such as those included in the Maguire^81^ niche concept^57^. More theoretical work is needed on the relationships between fuzzy set theory and niche theory. This would allow forecasting that is simultaneously practical and theoretically comprehensive and, thus, useful for conservation management and the scientific understanding of the biogeographic processes underlying the species response to climate changes.

## Methods

### Study area

The study area was the European, Asian, and African zones from 20° 00′ W to 60° 00′ E and from 09° 30′ N to 70° 00′ N (Fig. 1b). This area, which comprises the Western Palearctic and surrounding areas, fully covers the current breeding territories of the Atlas Long-legged Buzzard, as well as the western distribution of the Asian Long-legged Buzzard. Climatic heterogeneity is high, covering sub-tropical, desert, Mediterranean, Atlantic, and tundra climates^82–84^. To analyse the distribution of the subspecies, the study area was converted into 1-degree latitude × 1-degree longitude grid cells (*n* = 3839) using the *Create Fishnet* and *Intersect* tools from ArcGIS. These cells were used as operational geographic units (OGUs)^85,86^.

### Species distribution data

There is such little information on the Atlas Long-legged Buzzard that it is considered a gap in the knowledge of Western Palearctic birds^42^. In contrast to the Asian Long-legged Buzzard, it is mainly sedentary with some dispersal^38,58^. This medium-sized raptor occupies different habitat types in North Africa, occurring well into the Sahara^52^ and up to the Atlas Mountains, and breeding as high as *c* 2500 m above sea level^87^. It is relatively scarce in most of its breeding range, although it can be locally common in forest areas bordering open hunting grounds, such as the lakes area in the cedar forests of the Mid-Atlas^88^.

World atlases were used to obtain the distribution data to determine the breeding area of the Atlas Long-legged Buzzard^38,42,58^. This information was updated using the IUCN red list^89^, e-Bird (https://ebird.org/species/lolbuz1), and some personal records from Morocco and Spain^2^. We identified the OGUs where the species had been reported as breeding at least once from 1980 to 2020. Thus, we obtained a binary target variable representing breeding/not breeding at each OGU (breeding: n_1_ = 146, not breeding: n_0_ = 3693, Fig. 1b).

### Predictor variables and future scenarios

A set of 21 environmental variables (list and sources in Table 5) related to topography and climate between 1950 and 2000 were used in the biogeographic modelling procedure. These variables were digitized in raster format at a resolution of 1-km^2^ pixels. Values of these variables at each OGU were obtained by averaging the values of the 1-km^2^ pixels within them using the zonal function of ArcGIS 10.4.1 software.

**Table 5:** Variables selected to model the Atlas Long-legged Buzzard distribution grouped by environmental factor.

Code	Variable	Units	Source	*Exc*
**Topography**				
Alti	Altitude	m	(a)	–
Alti^2^	Altitude squared	m^2^	(b)	^c^
Slope	Slope	Degrees	(b)	^b^
**Climate**				
MeanTemp	Annual mean temperature	°C × 10	(c)	–
DiTempRange	Mean diurnal temperature range	°C × 10	(c)	–
Isoth	Isothermally	Percent	(c)	^a^
TempSeason	Temperature seasonality	Standard deviation	(c)	^b^
MaxTemp	Maximum temperature of warmest month	°C × 10	(c)	^a^
MinTemp	Minimum temperature of coldest month	°C × 10	(c)	^a^
TempAnRange	Temperature annual range	°C × 10	(c)	^c^
TempWetQ	Mean temperature of wettest quarter	°C × 10	(c)	–
TempDryQ	Mean temperature of driest quarter	°C × 10	(c)	^a^
TempWarmQ	Mean temperature of warmest quarter	°C × 10	(c)	^a^
TempColdQ	Mean temperature of coldest quarter	°C × 10	(c)	^c^
Prec	Annual precipitation	mm/year	(c)	–
PrecWetMonth	Precipitation of wettest month	mm/month	(c)	^a^
PrecDryMonth	Precipitation of driest month	mm/month	(c)	–
PrecSeason	Precipitation seasonality	coefficient of variation	(c)	^b^
PrecWetQ	Precipitation of wettest quarter	mm/quarter	(c)	^a^
PrecDryQ	Precipitation of driest quarter	mm/quarter	(c)	^a^
PrecWarmQ	Precipitation of warmest quarter	mm/quarter	(c)	^a^
PrecColdQ	Precipitation of coldest quarter	mm/quarter	(c)	^a^

Sources: (a) Ref.^116^; (b) calculated from *Alti* with ArcGIS software; (c) Ref.^90^. *Exc*. is the procedure that excluded the variable, being ^a^Spearman’s correlation value, ^b^FDR analysis and ^c^step-wise selection process.

Expected future values of the climatic variables were obtained for the periods 2041 to 2060 and 2061 to 2080^90^ (https://worldclim.org/). Four different Representative Concentration Pathways (RCP) were used to represent the extreme (2.6 and 8.5) and intermediate (6.0 and 4.5) trajectories of future CO_2_ emissions, with different effects on precipitation and temperature^7^. We also used two different Global Circulation Models (GCM; HadGEM2-ES and NorESM1-M) to consider other sources of uncertainty regarding the future climate of the study area^23,91,92^. This process resulted in eight sets of expected values of the climatic variables for each period of time.

### Model for the present

We performed a logistic regression of the binary target variable on each environmental variable separately. This is a supervised machine learning approach that assesses the predictive power of each variable according to the significance (α) of the score test of the corresponding regression. Multicollinearity between these variables was reduced by calculating the Spearman correlation coefficients *r* between them. For each pair of variables with *r* > 0.8, only the one with the highest individual predictive power was retained^93^. On the basis of this subset of predictors, the False Discovery Rate^94^ (FDR) was evaluated to control the increase in type I errors (i.e. familywise error rate), and therefore the likelihood of obtaining false significant results when a large number of variables are used in the modelling process^95^. Using the Benjamini and Yekutieli^96^ procedure for all forms of dependency between test statistics, only variables significantly associated with the distribution of the subspecies with α < 0.05 under an FDR value of 0.05 were accepted in subsequent modelling procedures.

A comprehensive model for the current probability of breeding at every OGU according to their climatic conditions was obtained by multivariate forward–backward stepwise logistic regression of the target variable on the remaining subset of predictors. This procedure starts with a model with no predictor variable (i.e. the null model), which yields a constant probability of breeding at each OGU equal to the prevalence of the OGUs where breeding was reported in the whole OGU dataset. Then, a significant combination of predictors (*y* or *logit*) is built by adding the variables that provide the most significant contribution to the model obtained in the previous step^51,97^. If no predictor variable significantly adds to the null model then no environmental model is produced. Variables with an overall, broad scale, predictive power are entered first in the modelling procedure, while those that add significant nuances to the previous model are entered in subsequent steps. To avoid redundancy, variables that do not significantly add to the predictive power of the final model are not included, as their effect, if any, is effectively included in the model via correlated variables.

The effect of prevalence on the resulting probability values was discounted to extract the pure response of the buzzard to environmental conditions^57,81^. This was done by obtaining favourability values (*F*) using the Eq. (1)^98^.

1 $$ F = \frac{{e^{y} }}{{\frac{{n_{1} }}{{n_{0} }} + e^{y} }} , $$

where *n*_1_ and *n*_0_ are the number of OGUs where breeding was reported or not reported, respectively, *e* is the Euler's number, and *y* is the logit resulting from the performed logistic regression.

Favourability values range from 0 (minimum favourability) to 1 (maximum favourability). A local favourability value of 0.5 indicates that the local probability of breeding of this subspecies is the same as its prevalence in the study area, i.e., is the probability expected by a null model unaffected by environmental predictors, where breeding is neither favoured nor unfavoured by the environment. Hence, favourability refers to the degree to which the environmental conditions favour the breeding of the subspecies^50,99^, being a favourability value of 0.5 the threshold separating favourable from unfavourable areas. The concept of favourability differs from that of suitability in: (1) there is only one way of obtaining favourability from probability and prevalence, while different algorithms yield idiosyncratic suitability values; (2) favourability values are interpretable in absolute terms, indicating the extent to which probability of local presence differs from that expected by chance in the whole sample, while suitability values are relative; and (3) favourability values represent the degree of membership of the localities to the fuzzy set of sites with conditions that are favourable for the species, which enables the application of fuzzy logic operations to distribution modelling^50^. However, given the continuous and fuzzy character of favourability^50^, the use of a favourability value of 0.5 as a cut-off point for crisply distinguishing favourable from unfavourable areas is not sufficiently informative^100^. Thus, we classified the areas into ‘high favourability’ (*F* ≥ 0.8), ‘intermediate favourability’ (0.2 < F < 0,8), ‘low favourability’ (0.00001 ≤ *F* ≤ 0.2), and ‘very-low favourability’ (*F* < 0.00001)^101^.

### Model assessment

The relative weight of each variable in the final model was assessed using the Wald test^102^. The discrimination capacity of the resulting model was evaluated using the Area Under the Receiver Operating characteristic Curve (AUC)^103,104^, which has an associated significance value. We used Cohen's Kappa Index to measure the degree to which the favourability of the OGUs with reported breeding or no reported breeding in the dataset was higher or lower than 0.5, respectively (Kappa is described as the proportion of specific agreement, whose values range from − 1 to + 1)^105^. As described by Fielding and Bell^106^ and Barbosa et al.^107^, we also applied a set of classification measures, whose values range from 0 to 1. These measures were sensitivity (the conditional probability of OGUs with reported breeding being classified as favourable), specificity (the conditional probability of OGUs with no reported breeding being classified as unfavourable), correct classification rate (CCR: the conditional probability of correctly classified OGUs), the over-prediction rate (OPR: the proportion of OGUs with no reported breeding in the area with favourability higher than 0.5), and the under-prediction rate (UPR: the proportion of OGUs with reported breeding in the area with favourability lower than 0.5). Good classification performance is shown by high Kappa, sensitivity, specificity, and CCR values and low over- and under-prediction rate values. Model calibration was assessed using the Hosmer–Lemeshow test^51^, where non-significant values (*p* > 0.05) indicate a good fit between predicted and observed probabilities (i.e. a well calibrated model). The test was performed by dividing the probability range of the model into 10 bins of equal range and checking that each bin included at least 15 OGUs and at least 5 OGUs in which reported breeding was expected in all of them, requirements that should be fulfilled for reliable model calibration^51^.

We evaluated the current potential for dispersion of the Atlas Long-Legged Buzzard using the potential change factor (Pch) in OGUs with breeding. This factor is the ratio between the OGUs with favourability value higher than 0.5 and OGUs with reported breeding. Values lower or higher than 1 indicate higher or lower potential regarding the actual breeding distribution of the buzzard, respectively^77^.

### Projection to future climate scenarios

Future climatic favourability values (*F*_*f*_) were obtained by replacing in the *logit* (*y*) of Eq. (1) the present values of the climatic variables with the expected future values according to each RCP and GCM and for each future period of time^23,108,109^. This process resulted in eight expected climatic favourability models for each period. An ensemble forecasting of the models was obtained for each period of time by calculating the mean values of the eight future climatic favourability models at each OGU. The uncertainty of the ensemble forecasting was computed using fuzzy set theory^43^, given that favourability values may be considered to be the degree of membership in the fuzzy set of areas favourable for buzzard breeding^98^. Thus, the favourability function is the membership function that assigns each OGU their degree of membership value^98^. The uncertainty of the ensemble forecasting at each OGU was computed as the difference at each OGU between the fuzzy union of the eight models (the maximum value of favourability of either of them at the OGU) and their fuzzy intersection (the minimum value of favourability of either of them at the OGU)^110^.

### Assessment of latitudinal variation

In biogeography, the barycentre (*B*) of a variable in the range of a species is the centre of gravity of a species distribution along the gradient of that variable^111–113^. When applied to geographic coordinates, the barycentre is indicative of the geographic centre of the distribution range^114^. The latitudinal barycentre of the OGUs with reported breeding of the Atlas Long-legged Buzzard (*B*_*bre*_) was obtained as the arithmetic mean of the latitudinal values at the centre of the OGUs. For the present and future models, the latitudinal barycentres of climatic favourability (*B*_*F*_) were obtained by weighting the latitude with the favourability using Eq. (2):

2 $$ B_{F} = \frac{{\sum \left( {La \times F} \right)}}{\sum F} , $$

where *La* and *F* are the latitude (in decimal degrees) and climatic favourability values, respectively, at each OGU. For the present model, the climatic favourability barycentre was applied to the following OGUs: (1) those with reported breeding of the buzzard (*B*_*FB*_), and (2) all those with reported breeding or otherwise within the longitudinal range where the subspecies was reported to breed (*B*_*FLo*_). The climatic favourability barycentre was also calculated using the future ensemble forecasting models for 2041 to 2060 (*B*_*F60*_) and 2061 to 2080 (*B*_*F80*_).

The difference between the geographic and climatic latitudinal barycentres in the OGUs where the buzzard was reported to breed (*B*_*bre*_ and *B*_*FB*_) represents the latitudinal disequilibrium (Ldis = *B*_*FB*_ − *B*_*bre*_) between current climatic favourability for breeding and the actual breeding areas. This latitudinal disequilibrium is northward if *B*_*FB*_ > *B*_*pres*_, southward if *B*_*FB*_ < *B*_*pres*_or in equilibrium if *B*_*FB*_ = *B*_*pres*_. The difference between the latitudinal climatic favourability barycentre at present inside the longitudinal breeding range and that forecast for the period 2041 to 2060 (*B*_*F60*_ −* B*_*FLo*_) was calculated to determine the latitudinal distance that the climatic favourability was forecasted to shift between the two periods. This shift is predicted to be northward if the barycentre for the future has a higher value than the present one. Similarly, we computed the difference between the latitudinal climatic favourability barycentres for the periods 2041 to 2060 and 2061 to 2080 (*B*_*F80*_ −* B*_*F60*_). We obtained two average rates of latitudinal climatic Favourability Displacement (FD_20–60_ and FD_60–80_, respectively) by dividing these distances by the number of years between the periods compared: that is, the years from 2020 until the end of the period 2041 to 2060 (40 years) and from 2060 until the end of the period 2061 to 2080 (20 years), respectively. These rates represent the distance that the climatic favourability is expected to shift latitudinally every year between each period of time, assuming gradual and constant shifts over time. Ldis and FD values were computed in latitudinal degrees and subsequently converted into kilometres considering the equivalence of the latitudinal degree at the equator (111.12 km).

### Fuzzy logic assessment of other changes in expected breeding distribution

Four fuzzy logic operations were used to forecast other impacts of climate change on Atlas Long-legged Buzzard climatic favourability^23^. These operations measure the increment in favourability (*I*), favourability overlap (*O*), favourability maintenance (*M*), and the predicted shift in favourability (*S*) in relation to the present^23^. Thus, they are collectively known as the IOMS framework (Eqs. 3–6)^115^:

3 $$ I = \frac{{c\left( {F_{f} } \right) - c\left( {F_{p} } \right)}}{{c\left( {F_{p} } \right)}} , $$

4 $$ O = \frac{{c\left( {F_{f} \cap F_{p} } \right)}}{{c\left( {F_{f} \cup F_{p} } \right)}} , $$

5 $$ M = \frac{{c\left( {F_{f} \cap F_{p} } \right)}}{{c\left( {F_{p} } \right)}}, $$

6 $$ S = \frac{{Min\left[ {c\left( {F_{p} } \right) - c\left( {F_{f} \cap F_{p} } \right), c\left( {F_{f} } \right) - c\left( {F_{f} \cap F_{p} } \right) } \right]}}{{c\left( {F_{p} } \right)}}, $$

where *c*(*X*) is the cardinality of the fuzzy set *X*: that is, the sum of the degrees of membership of all the OGUs (i.e. favourability values) in the fuzzy set *X*, which is a measure of the size of the fuzzy set; *Fp* and *Ff* are the fuzzy sets of present and future favourable areas for the buzzard, respectively; $F_{f} \cap F_{p}$ is the fuzzy intersection between future and present favourability defined by the minimum of the two favourability values in each OGU, representing the present climatic favourability conditions that are expected to persist in the future; $F_{f} \cup F_{p}$ is the fuzzy union between future and present favourability, defined by the maximum of the two favourability values in the OGU; and *Min* is the minimum of the two values in square brackets. Positive values in *I* indicate a gain in overall climatically favourable areas, whereas negative values indicate a net loss of climatic favourability. *O*, *M*, and *S* range from 0 to 1. *O* values closer to 1 are obtained when a large proportion of favourable areas are shared in the present and future models, whereas a value of zero indicates total separation between climatically favourable areas at present and in the future. An *M* value of 1 indicates that the present favourable areas will be completely maintained in the future projections. The lower the *M* values the less the currently favourable areas are going to remain climatically favourable. Values of *S* indicate the degree to which the loss of presently favourable areas is expected to be replaced by other new favourable areas elsewhere^53^.

## Supplementary information

Supplementary Information.
