# Supplementary material for: Fuzzy sets allow gaging the extent and rate of species range shift due to climate change
Source: Sci Rep. 2020 Oct 1;10:16272. doi: 10.1038/s41598-020-73509-y (PMC7530757; doi:10.1038/s41598-020-73509-y)

**Fuzzy sets allow gaging the extent and rate of species range shift due to climate change**

DARÍO CHAMORRO*, RAIMUNDO REAL & ANTONIO-ROMÁN MUÑOZ

Biogeography, Diversity, and Conservation Research Team, Dept. of Animal Biology, Science Faculty, Universidad de Málaga, E-29071, Malaga, Spain

* Corresponding author

E-mail addresses list:

Darío Chamorro: [dariochamorro@uma.es](mailto:dariochamorro@uma.es) ORCID: 0000-0003-4399-6998

Raimundo Real: [rrgimenez@uma.es](mailto:rrgimenez@uma.es) ORCID: 0000-0002-6642-1284

Antonio-Román Muñoz: [roman@uma.es](mailto:roman@uma.es) ORCID: 0000-0002-0253-7632

Running Title: Northwards shifting in favourable areas for African species

Obtained climatically favourability models for the Atlas long-legged Buzzard at each Representative Concentration Pathway (RCP) and Global Circulation Model (GCM) predicted to 2041-2060. Maps created using ArcMap (10.4.1) <https://desktop.arcgis.com/es/arcmap/>.


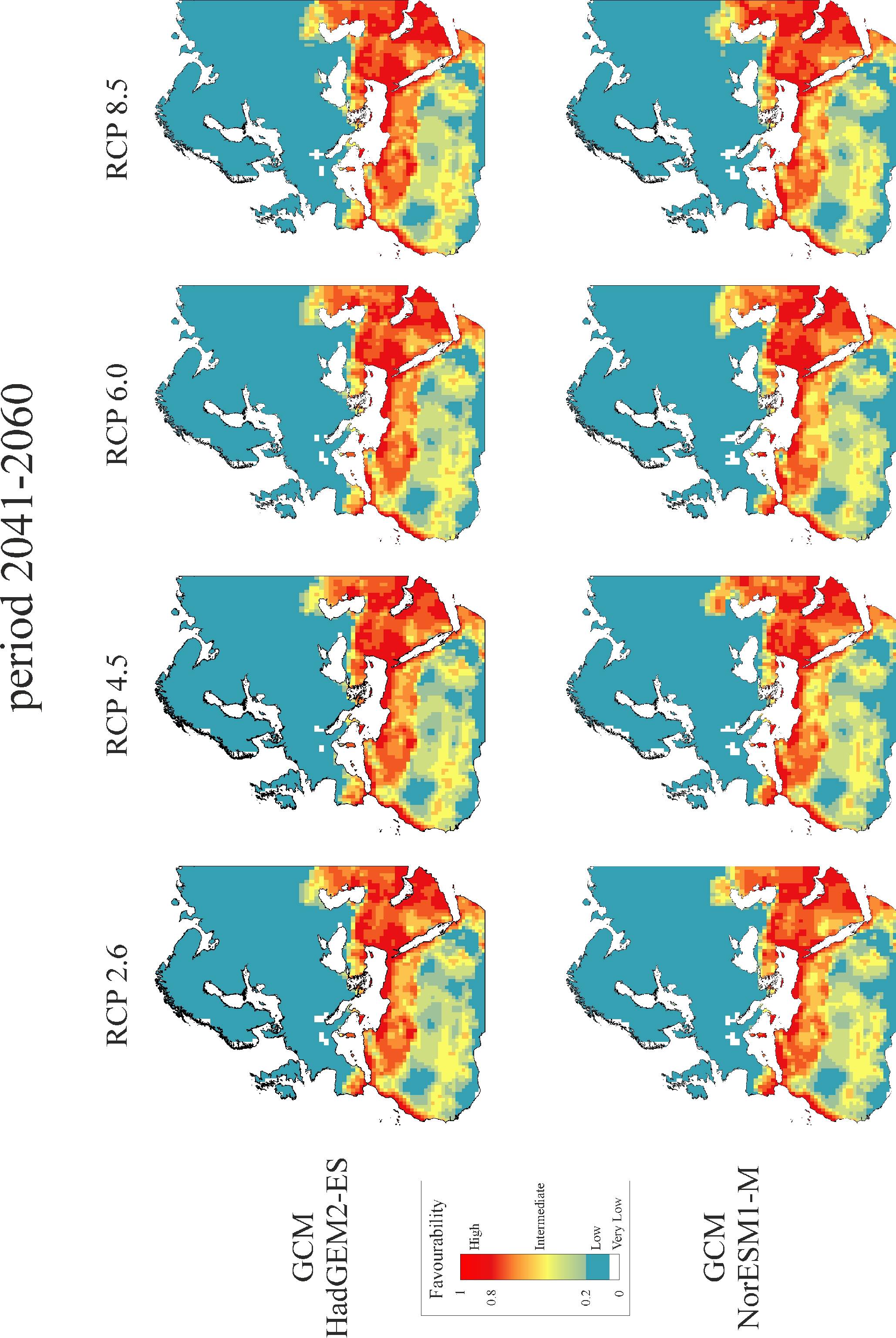


Obtained climatically favourability models for the Atlas long-legged Buzzard at each Representative Concentration Pathway (RCP) and Global Circulation Model (GCM) predicted to 2061-2080. Maps created using ArcMap (10.4.1) <https://desktop.arcgis.com/es/arcmap/>.


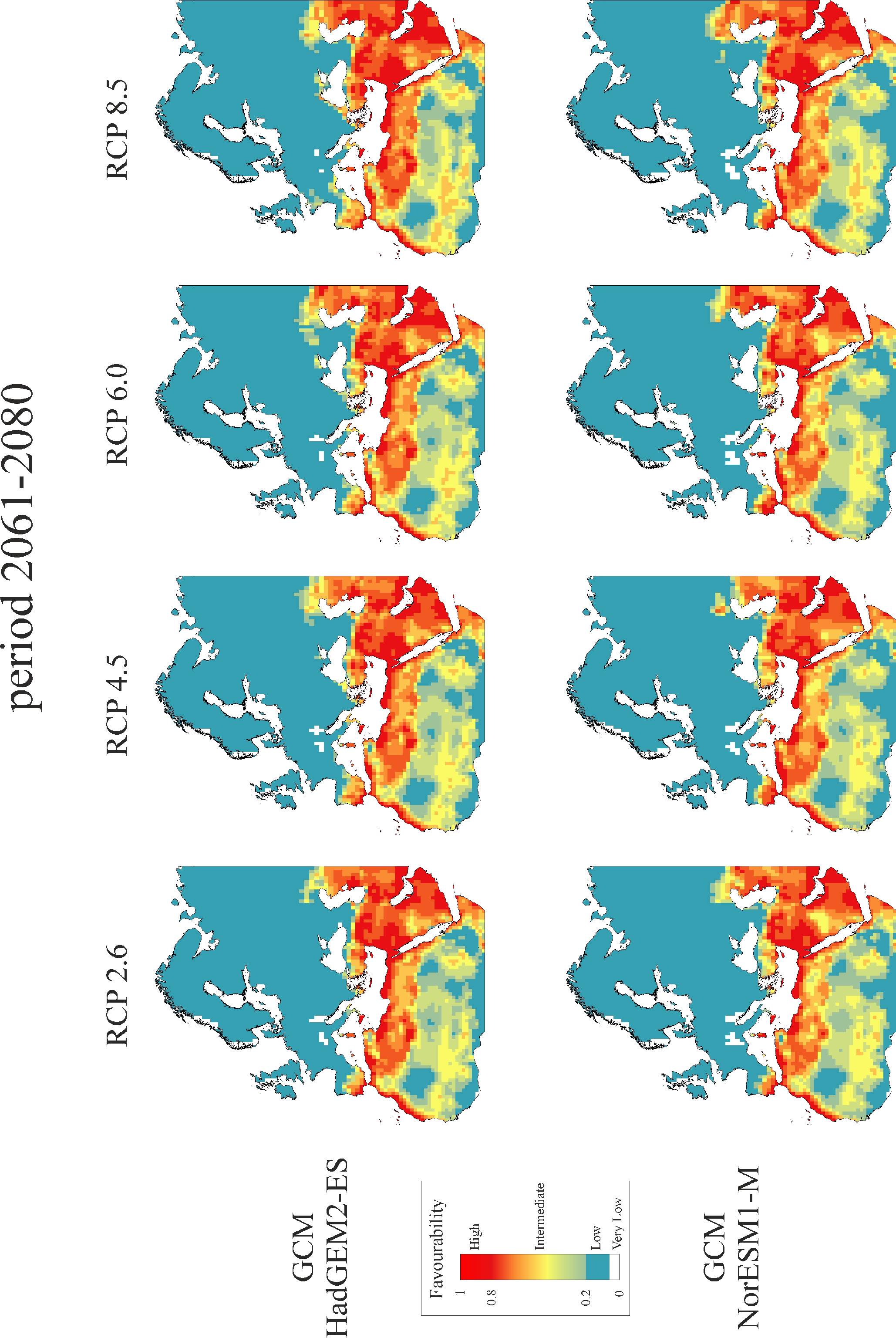

Supplement: Supplementary file 1 — Supplementary Information. [file 41598_2020_73509_MOESM1_ESM.docx]
